# Noninvasive Ventilation with Heliox for Respiratory Distress Syndrome in Preterm Infant: A Systematic Review and Meta-Analysis

**DOI:** 10.1155/2016/9092871

**Published:** 2016-11-22

**Authors:** Chen Long, Wang Li, Li Wanwei, Li Jie, Shi Yuan

**Affiliations:** ^1^Department of Pediatrics, Daping Hospital, Research Institute of Surgery, Third Military Medical University, Chongqing 400042, China; ^2^Department of Blood Transfusion, Daping Hospital, Research Institute of Surgery, Third Military Medical University, Chongqing 400042, China; ^3^Department of Obstetrics and Gynecology, The First Affiliated Hospital of Chongqing Medical University, Chongqing 400014, China

## Abstract

*Objectives.* To assess whether noninvasive ventilation with Heliox reduces the need for endotracheal ventilation and subsequent complications in preterm infants with respiratory distress syndrome (RDS).* Methods.* A search of major electronic databases, including MEDLINE and the Cochrane Central Register of Controlled Trials, for randomized or quasi-randomized controlled trials that compared noninvasive ventilation with Heliox versus noninvasive ventilation with standard gas for preterm infants with RDS was performed. The primary outcome was the incidence of intubation. The secondary outcomes were the level of PaCO_2_, the use of surfactant, and other complications.* Results.* Two randomized and one quasi-randomized controlled trials including 123 preterm infants were assessed. Heliox was found to significantly decrease the incidence of intubation (RR: 0.42; 95% CI: 0.23 to 0.78), the level of PaCO_2_ (MD: −9.61; 95% CI: −15.76 to −03.45), and the use of surfactant (RR: 0.25; 95% CI: 0.10 to 0.61) as compared with standard gas. No significant differences were found in other secondary outcomes.* Conclusions.* Noninvasive ventilation with Heliox decreases the incidence of intubation in preterm infants suffering from RDS. However, data on clinical outcomes are limited. Larger trials are needed to verify the beneficial effects.

## 1. Introduction

Respiratory distress syndrome (RDS) is a condition of respiratory distress which commences at or shortly after birth and increases in severity over the first three days of life, and it also is the most common cause of morbidity and mortality in preterm infants and is related inversely to the gestational age [1]. Endotracheal ventilation and exogenous surfactant replacement therapy are two standardized therapies to reduce neonatal mortality [2]. Despite improving survival [3], endotracheal ventilation is related to increasing risks of infection and ventilation-associated lung injuries. Importantly, prolonged duration of endotracheal ventilation induces a higher probability of death or survival with neurologic impairment and/or bronchopulmonary dysplasia (BPD) in the postneonatal period [4]. There is thus a trend to minimize the use of mechanical ventilation.

To this day, early use of noninvasive respiratory support is the most effective pathway to reduce these risks above. However, noninvasive ventilation strategies are only partly helpful, as about 10.5%–50% fail and need endotracheal ventilation [5]. Since 1935, the use of Heliox (79% helium and 21% oxygen) has been proposed as a standard therapy for severe asthma, acute upper airway obstruction [6]. Helium is an inert, colorless, and odorless gas and has very low density, and when the nitrogen in inspired standard air is replaced with helium, the density of mixture is 3 times less than standard air [7]. Studies have reported beneficial effects such as the reduction of lung inflammation, flow turbulence, and work of breathing and air-trapping and the improvement of the distal-airway transmission of aerosol particles [8], and the effects of Heliox have been attributed to the physical characteristics of helium. Recently, noninvasive ventilation strategies with Heliox have been used for the purposes of minimizing physical and chemical injuries, as well as supporting adequate gas exchange in some RCTs and non-RCTs for preterm neonates with RDS, but the clinical application was rare and the results remained inconsistent.

The objective of this systematic review was to evaluate whether noninvasive ventilation with Heliox would reduce the requirement for endotracheal ventilation and subsequent complications in preterm infants with RDS as compared with standard gas.

## 2. Methods

Studies were added to the review whether they were randomized or quasi-randomized controlled trials. The interventions for comparison were Heliox and standard gas in preterm infants with RDS and supported by noninvasive ventilation. We did not put restrictions on studies as to language.

The search strategies and assessment methods are similar to our previous study [5]. A systematic literature search was conducted in March 2016, using the methods of the Cochrane Collaboration for Systematic Reviews of Interventions [9]. The databases searched included MEDLINE (1980 to March 2016) and the Cochrane Central Register of Controlled Trials (all years). The keywords “nasal intermittent positive pressure ventilation (NIPPV)” or “nasal continuous positive airway pressure (CPAP)” or “bi-level positive airway pressure (BiPAP)” or “noninvasive positive pressure ventilation” and “preterm” or “premature” or “neonate” and “respiratory distress syndrome (RDS)” and “heliox” or “helium/oxygen” were used. Meantime, the search was limited to human studies. We applied the Cochrane sensitivity-maximizing and Cochrane sensitivity- and precision-maximizing strategies as our special search strategies [9]. The criteria for a trial to be included in the meta-analysis were as follows: (1) trial involving preterm infants with RDS and (2) trial comparing noninvasive ventilation with Heliox and standard gas.

The studies obtained through the search strategies described above were imported to an electronic bibliographic management program. We reviewed the titles and abstracts of the remaining articles and excluded those that were not related to our topic and those that did not meet the eligibility criteria. The full-text versions were obtained for the relevant articles that could be included in the review.

The research strategies, article-extracting, and data analysis were performed independently by three reviewers. Data analysis included study design, study interventions, number of subjects in each group, demographic characteristics, inclusion and exclusion criteria, primary and secondary outcomes, and variables used to assess study quality.

The primary outcome was the need for intubation, and the observation time was any time before discharge. The secondary outcomes were the level of PaCO_2_ at the time Heliox ceased, the use of surfactant and subsequent complications, including the incidences of BPD, intraventricular hemorrhage (IVH) of any grade, necrotizing enterocolitis (NEC), retinopathy of prematurity (ROP), patent ductus arteriosus (PDA), and periventricular leukomalacia (PVL), total time of noninvasive ventilation, duration of hospitalization, and death before hospital discharge.

The Cochrane Risk of Bias tool [9] was applied to assess the methodological quality of the included studies. Discrepancies between the three reviewers were resolved through discussion (Table 3). Meta-analysis was performed using version 5.2 of Review Manager. To assess heterogeneity, 2 distribution and Higgins *I* ^2^ statistics were calculated to determine the percentage of total variation across studies resulting from heterogeneity. *I* ^2^ statistics approximating 25%, 50%, and 75% were considered low, medium, and high heterogeneity, respectively. The fixed-effects models were present, and the random-effects models were used whenever considerable heterogeneity was shown. For categorical data, the effect is expressed as the RR, and for continuous data the effect is expressed as the weighted mean difference (95% CI).

## 3. Results

### 3.1. Description of Studies

**Figure 1:**
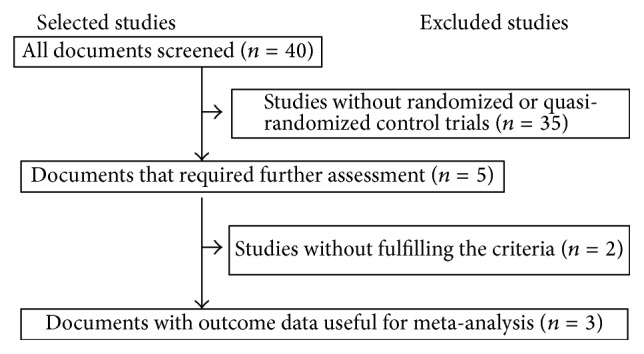
The selection course of the included papers.

Forty studies were identified, of which thirty-five were excluded because they were not RCTs or quasi-RCTs. Five trials underwent further evaluation, and two were excluded because they did not meet the inclusion criteria. Three eligible studies were included in the final analysis [10–12] (Figure 1).

Tables 1 [Table tab2]–3 summarized the characteristics and quality assessments of these studies. These studies were conducted in Italy and China. A total of 123 infants were enrolled in the three studies. Two studies were RCTs and one study was quasi-RCT.

### 3.2. Primary Outcomes

**Figure 2:**
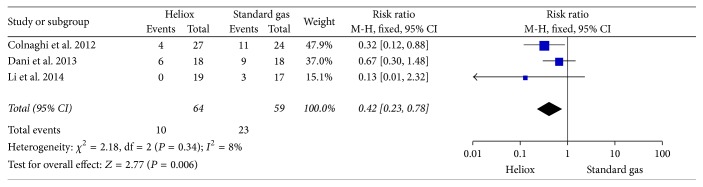
The comparison of Heliox versus standard air for the incidence of intubation.

Each study reported the requirement for intubation and mechanical ventilation. The meta-analysis estimated a significant decrease for the need for invasive ventilation in the Heliox group as compared with the standard gas group (RR: 0.42; 95% CI: 0.23–0.78) in the fixed-effects model (Figure 2). Heterogeneity was not found among the 3 trials (*P* = 0.34, *I* ^2^ = 8%).

### 3.3. Secondary Outcomes

**Figure 3:**
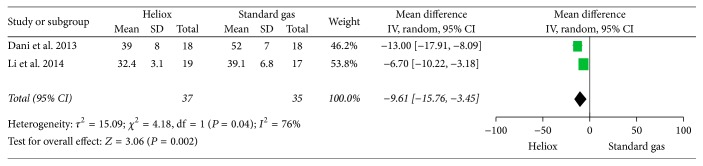
The comparison of Heliox versus standard air for the level of PaCO_2_.

Data for the secondary outcome demonstrated a significant decrease for the level of PaCO_2_ in the Heliox group (mean difference: −9.61; 95% CI: −15.76–−3.45), with heterogeneity among the two trials (*P* = 0.04, *I* ^2^ = 76%) (Figure 3).

**Figure 4:**
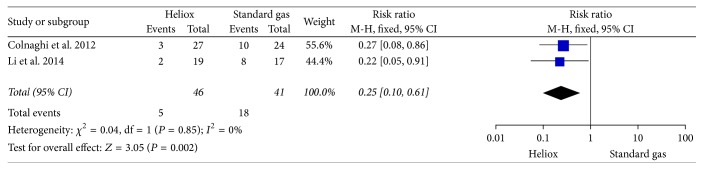
The comparison of Heliox versus standard air for the use of surfactant.

Data also demonstrated a significant decrease for the use of surfactant in the Heliox group (RR: 0.25; 95% CI: 0.10–0.61), without heterogeneity among the two included trials (*P* = 0.85, *I* ^2^ = 0%) (Figure 4).

No significant differences were found in other secondary outcomes of included studies between the two groups (Table 4).

## 4. Discussion

In the present meta-analysis involving three RCTs, we aimed to assess the rate of endotracheal intubation and subsequent complications in preterm infants with RDS through comparing noninvasive ventilation with Heliox and standard gas. The results showed a significant decrease for the need of endotracheal intubation in the Heliox group as compared with the standard gas group. Similarities also appeared in the clearance of PaCO_2_ and the use of surfactant. These findings suggest that Heliox does increase the beneficial effects of noninvasive ventilation and contribute to a reduced risk of endotracheal ventilation in preterm infants with noninvasive ventilation.

Previous studies have demonstrated the beneficial effects of Heliox compared with standard gas in preterm infants. Specifically, Heliox has been shown to significantly reduce the requirement for ventilatory support and improve gas exchange [13–15]. A recent meta-analysis found that infants treated with Heliox had a significantly lower mean clinical respiratory score in the first hour after starting treatment when compared to those treated with air or oxygen [16]. And these results were consistent with the present meta-analysis. However, there were significant heterogeneities. One of the causes of heterogeneities might be the observation time of intervention. Among the trials included, the observation time of “need for mechanical ventilation” was different. The observation time of “failure of Heliox/standard gas” in the study by Dani et al. [11] was “during the 24 hours following extubation” and it was “within the first 7 days of life” in the study by Colnaghi et al. [12]. But the study by Li et al. [10] did not limit the observation time.

Although basic mechanisms by which Heliox improves efficacy are clear, a better understanding of its exact actions is needed. The possible mechanisms by which Heliox works are decreasing mean airway resistance and respiratory work, as well as improving gas exchange and lung compliance. Interestingly, Heliox might also have the potential for chemical benefits as an inert gas. The included RCT of Li et al. [10] showed that Heliox significantly reduced mean length of ventilation in comparison to standard gas, and the latter was positively correlated with interleukin-6 at baseline (*r* = 0.474, *P* = 0.006). Compared to animals ventilated with standard gas, levels of interleukin-8 and myeloperoxidase were also lower in animals ventilated with Heliox [8].

Prophylactic, early, and enough surfactant replacement therapy has been reported to reduce effectively the incidence of intubation and complications in preterm infants with RDS as compared with later selective surfactant administration [17]. However, the INSURE (intubation-surfactant-extubation) technique of surfactant administration is an invasive operation, and it is not successful in all preterm neonates with RDS, with a reported failure rate ranging from 19 to 69%. And the unsuccessful INSURE technique required subsequent intratracheal ventilation [18]. In our review with meta-analysis from two trials of Li et al. [10] and Colnaghi et al. [12], a remarkable decrease was demonstrated for the need of surfactant in the group of infants who received Heliox, and the difference was statistically significant. Our results further confirmed that Heliox was more successful than standard gas in preventing the INSURE-associated endotracheal intubation in the initial treatment of premature infants with RDS. Noninvasive respiratory support and Heliox therapy may have synergistic effects on uniform distribution of oxygen and carbon dioxide, as well as decreasing alveolar surface tension. With the optimal lung capacity, relatively constant airway, and alveolar pressure, the pulmonary gas distribution at a uniform state could cause maximally less alveolar excessive expansion or atelectasis and, hence, avoid injury of lung.

BPD is a complex disorder and remains the most common complication of very preterm infants [19]. Initiation and/or maintenance of endotracheal ventilation, especially during the first week of life, may activate the alveolar macrophages, leading to the release of proinflammatory cytokines. Exposure to oxygen with high concentrations actually also potentiates the inflammatory cascade. Moreover, ventilator-associated lung injuries may lead to the ongoing inflammation and oxidative stress in the lung, finally leading to BPD. Many studies have been done to compare the effects between Heliox and standard gas on BPD and the incidence of BPD. Szczapa et al. [20] reported that mechanical ventilation with Heliox resulted in the improvement of respiratory function and oxygenation in infants with severe BPD requiring mechanical ventilation. Wolfson et al. [21] also indicated that Heliox decreased the work of breathing and airway resistance and reduced respiratory muscle fatigue and caloric requirements for breathing, thus providing additional calories for growth and recovery. In our meta-analysis, pooling of data from the two trials of Dani et al. [11] and Colnaghi et al. [12] did not reveal the beneficial effects for decreasing the incidence of BPD as compared with standard gas. Although a similar result was found in the study by Elleau et al. [15], the latter should be reconsidered because the sample size of this study was small and it was reported in the presurfactant era.

In addition, our review also revealed that Heliox was related to the reduction of time of noninvasive ventilation. No heterogeneity has been found [10, 12].

Our review from two trials [11, 12] showed that Heliox could not shorten the duration of hospitalization as compared with the standard gas. Besides, Heliox did not show any benefit in decreasing the incidence of PDA, ROP, BPD, and NEC. No heterogeneity has been found among the trials.

Furthermore, several modes of noninvasive respiratory support were used in the three included trials, including NIPPV, CPAP, and BiPAP. Up to now, numerous studies and meta-analyses have compared the effects of noninvasive ventilation on the incidence of intubation and subsequent complications, and the results remained inconsistent [5, 22–25]. Therefore, the results of the meta-analysis could be affected by the selection of noninvasive ventilation strategies.

In our review from three trials [10–12], the times of Heliox administration were different, with 3 hours by Li et al. [10], 24 hours by Dani et al. [11], and 12 hours by Colnaghi et al. [12]. As Martinón-Torres [26] said, one important advantage (and disadvantage) of Heliox is that it works only while being administered, and some beneficial effects of Heliox can be noted soon after initiation for that particular patient. In contrast, once Heliox is withdrawn, the symptoms could be aggravated [20]. Besides the short-term effects in preterm infants, neonatologists are more concerned with the long-term benefits, especially in very preterm infants. Therefore, the optimal beneficial time of Heliox administration is unclear and more trials are needed to verify it. Conclusions should be cautious because of the significant heterogeneity of administration time of Heliox among the studies. Szczapa et al. [20] proposed a question of how long Heliox should be continued and what should be set as the criteria for stopping it. One explanation was that Heliox should be continued during BPD exacerbation in order to minimize further lung injury associated with mechanical ventilation and stopped when lung function improved. The administration time of Heliox might be determined by the aim of used Heliox. For minimizing intubation in primary respiratory support, Heliox might be used for twelve to seventy-two hours [12], but, for avoiding reintubation and reducing the incidence of BPD, continued Heliox might be needed for more than eight days [15].

One important cause to explain the inconsistence among the included studies might be gestational age. In our review from three trials [10–12], the mean gestational ages were different, with 34.2 weeks by Li et al. [10], 25.4 weeks by Dani et al. [11], and 30.6 weeks by Colnaghi et al. [12]. Nowadays, preterm infants were actually divided into late preterm (34–36 weeks), moderate preterm (32-33 weeks), and very preterm (<32 weeks). In the very preterm infants, the incidence rate of RDS gradually has been confirmed to be increased with decreasing gestational age. EuroNeoStat figures for 2006 showed an incidence of 92% at 24-25 weeks, 88% at 26-27 weeks, 76% at 28-29 weeks, and 57% at 30-31 weeks of gestational age [1]. In the infants with gestational age less than 30 weeks, an obvious increase was observed in the incidence rate of RDS. It might therefore be improper to conduct the analysis in preterm infants with long time span, and preterm birth should be also divided into more subgroups according to the gestational age, such as 30–32 weeks, 28–32 weeks, and 26–28 weeks. Similarities also appeared in the complications of the secondary outcomes, and this was a main limitation in the analysis.

There were inconsistent results about side effects of Heliox administration in the previous studies. Szczapa et al. [20] indicated that mechanical ventilation with Heliox was feasible and could be applied without side effects in preterm infants with severe BPD. Spontaneously breathing Heliox could be tolerated in preterm infants with BPD [21]. Moreover, no side effects appeared even after eight days of administration of Heliox in preterm infants with RDS [15]. The above studies were in agreement with the comment of Martinón-Torres [26], in which no lines of evidence of harmful effects of Heliox were reported in 73 clinical trials. In the present review, there were also no side effects of Heliox in the three included trials. Actually, as far as the properties of helium are concerned, no side effects are a reasonable speculation. In contrast, several studies suggested side effects of Heliox. In a preliminary study designed to assess the tolerance to Heliox in infants with BPD, spontaneously breathing Heliox had immediate consequences such as wakening, crying, decrease in skin temperature, and hypoxia [27]. Similarly, hypoxia was also reported by Butt et al. [28]. More studies are needed to observe the possible side effects of Heliox. Therefore, more trials are also needed to verify them in the future.

Last but not least, the relatively high costs of Heliox administration should be considered [29]. Among the included trials, the average cost was similar. The cost of Heliox of “12 hours” was “EUR750” in the study by Colnaghi et al. [12] and that of “3 hours” was “EUR200” in the studies by Li et al. [10]. Possibly, recycled use of Heliox may be a better selection and further direction.

The major limitation of the present study was the small sample size, and trials with small sample size were more likely to show larger beneficial effects than trials with large sample size [30]. And these beneficial effects were consistent with the reports of Zhang et al. [31] and Papageorgiou et al. [32]. The authors thought that it might be due to the lower methodological quality in small trials. As far as we are concerned, the cause of inducing the differences between small trials and large trials might be the baseline differences of the included patients. An example was when the pregnancy-associated diseases of mothers were balanced completely; the results of the small sample trial [33] were consistent with the multicenter trial [24]. These problems could be overcome in additional multicenter studies with large sample size or more strict inclusion criteria in the small trials. Given the potential limitations, more trials are needed in the future.

## 5. Conclusions

In summary, the present study supports the updated lines of evidence. Based on the results, the present review provides several lines of evidence that noninvasive ventilation with Heliox is more successful than noninvasive ventilation with standard gas in avoiding invasive ventilation, when used for the treatment of preterm infants with RDS. However, it is also clear that data on clinical outcomes are limited. Therefore, any formal grading at this time is improper. Given these important limitations, further trials are needed to assess the use of Heliox.

## Figures and Tables

**Table 1:** The characteristics of included papers.

	N (*n*)		Gestational age (weeks)		Birth weight (g)		Male	
	Heliox	Standard air	Heliox	Standard air	Heliox	Standard air	Heliox	Standard air
Li et al. 2014	19	17	34.2 ± 1.8	34.3 ± 1.8	2150 ± 470	2190 ± 440	13	10
Dani et al. 2013	18	18	25.4 ± 1.5	25.8 ± 1.9	680 ± 150	750 ± 190	10	7
Colnaghi et al. 2012	27	24	30.6 ± 1.4	30.6 ± 1.2	1454.0 ± 332.2	1430.3 ± 327.4	18	15

**Table 2 tab2:** Details of included papers.

	Li et al. 2014	Dani et al. 2013	Colnaghi et al. 2012
Single or multicenter design	Single	Single	Multicenter
Mode of noninvasive ventilation	NIPPV	NCPAP or BiPAP	NCPAP
Time of Heliox administration (hours)	3	24	12
Heliox expenditure (¥/infant)	2000	—	7500
Whether or not surfactant was given	Surfactant was given only as rescue therapy	Early rescue surfactant treatment when FiO_2_ > 0.30	Surfactant was given only as rescue therapy
Whether or not noninvasive ventilation was used as primary support	Yes	No	Yes
Side effects	No	No	No

Exchange rate in 1/1/2008: 1¥ = 0.1€.

**Table 3:** Bias assessment of included papers.

	Li et al. 2014	Dani et al. 2013	Colnaghi et al. 2012
Allocation concealment	Yes	No	Yes
Sequence generation	Yes	No	Yes
Blinding (participants)	Unclear	Unclear	Unclear
Blinding (outcome assessors)	Yes	Unclear	Yes
Incomplete data address	Yes	Yes	Yes
Free of selective reporting	Yes	Yes	Yes
Free of other biases	No	Unclear	Unclear

**Table 4:** Pooled estimates for Heliox.

Secondary outcomes	Heliox versus standard gas								
	Colnaghi et al. 2012		Dani et al. 2013		Li et al. 2014		RR/mean difference (95% CI)	Heterogeneity	
	27	24	18	18	19	17		*P* value	*I* ^2^
Incidence of bronchopulmonary dysplasia	5	3	7	11	0	0	0.81 [0.38–1.73]	0.25	23%
Incidence of patent ductus arteriosus	12	10	16	16	7	5	1.06 [0.79–1.43]	0.82	0%
Incidence of retinopathy of prematurity	1	1	4	5	0	0	0.82 [0.28–2.34]	0.94	0%
Incidence of necrotizing enterocolitis	0	1	2	3	3	1	0.94 [0.30–2.91]	0.45	0%
Hospital stay (days)	52 ± 30	47 ± 33	115 ± 18	109 ± 15	—	—	5.78 [−3.06–14.63]	0.20	0%
Time of noninvasive ventilation (days)	26 ± 37	33 ± 6	—	—	1.6 ± 0.6	2.5 ± 1.0	−0.91 [−1.46–−0.36]	0.001	0%
Incidence of intraventricular hemorrhage	0	0	5	4	0	0	1.25 [0.40–3.91]	Not applicable	
Incidence of periventricular leukomalacia	0	0	2	1	0	0	2.00 [0.20–20.15]	Not applicable	
Death	0	0	3	2	0	0	1.50 [0.28–7.93]	Not applicable	

## Abbreviations

RDS: Respiratory distress syndrome
BPD: Bronchopulmonary dysplasia
PLV: Periventricular leukomalacia
IVH: Intraventricular hemorrhage
PDA: Patent ductus arteriosus
NEC: Necrotizing enterocolitis
ROP: Retinopathy of prematurity.

## References

[1] Sweet D. G., Carnielli V., Greisen G. (2013). European consensus guidelines on the management of neonatal respiratory distress syndrome in preterm infants—2013 update. *Neonatology*.

[2] St Clair C., Norwitz E. R., Woensdregt K. (2008). The probability of neonatal respiratory distress syndrome as a function of gestational age and lecithin/sphingomyelin ratio. *American Journal of Perinatology*.

[3] Stoll B. J., Hansen N. I., Bell E. F. (2010). Neonatal outcomes of extremely preterm infants from the NICHD Neonatal Research Network. *Pediatrics*.

[4] Schmidt B., Asztalos E. V., Roberts R. S., Robertson C. M. T., Sauve R. S., Whitfield M. F. (2003). Impact of bronchopulmonary dysplasia, brain injury, and severe retinopathy on the outcome of extremely low-birth-weight infants at 18 months: results from the trial of indomethacin prophylaxis in preterms. *Journal of the American Medical Association*.

[5] Li W., Long C., Zhangxue H. (2015). Nasal intermittent positive pressure ventilation versus nasal continuous positive airway pressure for preterm infants with respiratory distress syndrome: a meta-analysis and up-date. *Pediatric Pulmonology*.

[6] Barach A. (1935). The use of helium in the treatment of asthma and obstructive lesions in the larynx and trachea. *Annals of Internal Medicine*.

[7] Martinón Torres F., Martinón Sánchez J. M., Casado Flores J. (2011). Heliox therapy. *Mechanical Ventilation in Newborns, Infants and Children*.

[8] Nawab U. S., Touch S. M., Irwin-Sherman T. (2005). Heliox attenuates lung inflammation and structural alterations in acute lung injury. *Pediatric Pulmonology*.

[9] Higgins J. P. T., Green S. (2006). *Cochrane Handbook for Systematic Reviews of Interventions Version 4.2.6*.

[10] Li X., Shen J., Zhao J., Tang S., Shi Y. (2014). Nasal intermittent positive pressure ventilation with heliox in premature infants with respiratory distress syndrome: a randomized controlled trial. *Indian Pediatrics*.

[11] Dani C., Fontanelli G., Lori I., Favelli F., Poggi C. (2013). Heliox non-invasive ventilation for preventing extubation failure in preterm infants. *Journal of Maternal-Fetal and Neonatal Medicine*.

[12] Colnaghi M., Pierro M., Migliori C. (2012). Nasal continuous positive airway pressure with heliox in preterm infants with respiratory distress syndrome. *Pediatrics*.

[13] Migliori C., Gancia P., Garzoli E., Spinoni V., Chirico G. (2009). The effects of helium/oxygen mixture (heliox) before and after extubation in long-term mechanically ventilated very low birth weight infants. *Pediatrics*.

[14] Stucki P., Scalfaro P., de Halleux Q., Vermeulen F., Rappaz I., Cotting J. (2002). Successful management of severe respiratory failure combining heliox with noninvasive high-frequency percussive ventilation. *Critical Care Medicine*.

[15] Elleau C., Galperine R.-I., Guenard H., Demarquez J.-L. (1993). Helium-oxygen mixture in respiratory distress syndrome: a double-blind study. *The Journal of Pediatrics*.

[16] Liet J.-M., Ducruet T., Gupta V., Cambonie G. (2015). Heliox inhalation therapy for bronchiolitis in infants. *Cochrane Database of Systematic Reviews*.

[17] Stevens T. P., Harrington E. W., Blennow M., Soll R. F. (2007). Early surfactant administration with brief ventilation vs. selective surfactant and continued mechanical ventilation for preterm infants with or at risk for respiratory distress syndrome. *Cochrane Database of Systematic Reviews*.

[18] Vento G., Pastorino R., Boni L., Cota F., Carnielli V., Cools F. (2016). Efficacy of a new technique—INtubate-RECruit-SURfactant-Extubate—‘IN-REC-SUR-E’—in preterm neonates with respiratory distress syndrome: study protocol for a randomized controlled trial. *Trials*.

[19] Jobe A. H. (2011). The new bronchopulmonary dysplasia. *Current Opinion in Pediatrics*.

[20] Szczapa T., Gadzinowski J., Moczko J., Merritt T. A. (2014). Heliox for mechanically ventilated newborns with bronchopulmonary dysplasia. *Archives of Disease in Childhood—Fetal and Neonatal Edition*.

[21] Wolfson M. R., Bhutani V. K., Shaffer T. H., Bowen F. W. (1984). Mechanics and energetics of breathing helium in infants with bronchopulmonary dysplasia. *The Journal of Pediatrics*.

[22] Shi Y., Tang S., Zhao J., Shen J. (2014). A prospective, randomized, controlled study of NIPPV versus nCPAP in preterm and term infants with respiratory distress syndrome. *Pediatric Pulmonology*.

[23] Meneses J., Bhandari V., Alves J. G., Herrmann D. (2011). Noninvasive ventilation for respiratory distress syndrome: a randomized controlled trial. *Pediatrics*.

[24] Kirpalani H., Millar D., Lemyre B., Yoder B. A., Chiu A., Roberts R. S. (2013). A trial comparing noninvasive ventilation strategies in preterm infants. *The New England Journal of Medicine*.

[25] Meneses J., Bhandari V., Alves J. G. (2012). Nasal intermittent positive-pressure ventilation vs nasal continuous positive airway pressure for preterm infants with respiratory distress syndrome: a systematic review and meta-analysis. *Archives of Pediatrics and Adolescent Medicine*.

[26] Martinón-Torres F. (2015). What's weighing down heliox?. *The Lancet Respiratory Medicine*.

[27] De Gamarra E., Moriette G., Farhat M., Walti H. (1998). Heliox tolerance in spontaneously breathing neonates with bronchopulmonary dysplasia. *Biology of the Neonate*.

[28] Butt W. W., Koren G., England S. (1985). Hypoxia associated with helium-oxygen therapy in neonates. *The Journal of Pediatrics*.

[29] Valli G., Paoletti P., Savi D., Martolini D., Palange P. (2007). Clinical use of heliox in asthma and COPD. *Monaldi Archives for Chest Disease—Pulmonary Series*.

[30] Sterne J. A. C., Egger M. (2001). Funnel plots for detecting bias in meta-analysis: guidelines on choice of axis. *Journal of Clinical Epidemiology*.

[31] Zhang Z., Xu X., Ni H. (2013). Small studies may overestimate the effect sizes in critical care meta-analyses: a meta-epidemiological study. *Critical Care*.

[32] Papageorgiou S. N., Antonoglou G. N., Tsiranidou E., Jepsen S., Jäger A. (2014). Bias and small-study effects influence treatment effect estimates: a meta-epidemiological study in oral medicine. *Journal of Clinical Epidemiology*.

[33] Chen L., Wang L., Li J., Wang N., Shi Y. (2015). Noninvasive ventilation for preterm twin neonates with respiratory distress syndrome: a randomized controlled trial. *Scientific Reports*.

